# Mortality audit in general surgery unit and lessons learned at a Nigerian tertiary hospital: a single centre observational study

**DOI:** 10.11604/pamj.2022.41.228.29075

**Published:** 2022-03-18

**Authors:** Aloysius Ugwu-Olisa Ogbuanya, Vincent Chidi Enemuo, Uche Emmanuel Eni, Chinedu Gregory Nwigwe, Onyeyirichi Otu

**Affiliations:** 1 Department of Surgery, Alex Ekwueme Federal University Teaching Hospital, Abakaliki (AEFUTHA), Ebonyi State, Nigeria,; 2 Department of Surgery, Ebonyi State University, Abakaliki (EBSU), Ebonyi State, Nigeria,; 3 Department of Surgery, University of Nigeria, Nsukka (UNN), Nigeria

**Keywords:** Emergency, general surgery, laparotomy, mortality, peritonitis

## Abstract

**Introduction:**

mortality among surgical admissions is a global phenomenon, but the rates, pattern and factors that predict such deaths vary from region to region and even in one region, it varies among institutions. The aim was to document the pattern and factors that influence mortality in the general surgery unit of our institution.

**Methods:**

this was a seven-year retrospective, case-control study. All general surgery admissions managed at Alex Ekwueme Federal University Teaching Hospital, Abakaliki, Nigeria from January 2013 to December 2019 were included. Data were retrieved from case files of those managed during the period. Pattern and factors associated with increased mortality were analyzed and presented in tabular and descriptive forms.

**Results:**

of 4,898 general surgery admissions, 481 deaths were recorded, giving a crude mortality rate of 9.8%. Though highest number of deaths occurred in those in the 16-45 years age range, crude mortality rate was highest in elderly patients (>65 years). Generalized peritonitis was the most common cause of death, representing 38.9% of all deaths followed by cancers (22.9%), then abdominal injuries (16.8%). Of the 110 deaths from cancers, breast cancer (40, 36.4%) was the most important cause followed by colorectal cancers (29, 26.4%). Overall, 78.2% of the deaths occurred in emergency cases. In the logistic regression analysis, the following were significantly associated with mortality: advanced age, comorbidities, emergency presentation, high ASA scores (III-V) and delayed presentation.

**Conclusion:**

significant mortality occurs in our general surgery unit and is higher in older patients, and in those with generalized peritonitis, abdominal trauma and cancers.

## Introduction

Remarkable gains have been made in global health in the past 25 years, but progress has not been similar between high human development index (HDI) countries and low HDI nations [1-3]. Despite significant progress made by the industrialized nations on the areas of human and material resources, development of safe, essential, life-saving surgical and anaesthesia care in low- and medium-income countries (LMICs) has stagnated or regressed [2,4,5].

Indeed, it has been reported that mortality from anaesthesia and surgery in many countries in sub-Saharan Africa remains at levels last seen in high income countries 70 years ago [5]. Recent published data suggest that about 1 in 50,000 to 1 in 200,000 anaesthesia-related deaths occur in high income countries, but in LMICs, the rates are reportedly 100 to 1,000 times higher [3,5]. Consequently, the World Health Organization (WHO), through a patient safety program referred to as “safe surgery saves lives” developed a surgical safety checklist (SSC) as a means of improving the safety of surgical care around the world [5-7]. In its classic form, it represents a 19-item checklist program that emphasizes on performing safety checks and good team communication at various stages in the perioperative period to reduce adverse perioperative events [5,6]. In a multinational study involving eight hospitals from diverse economic settings, its use improved compliance with standards of care by 65% and reduced death rates following surgery by nearly 50.0% [5,6]. Mortality following operative surgery is a global phenomenon and occurs in industrialized and developing nations, rural and urban setting, elective and emergency set up and in wealthy and poor patients. However, its immediate cause varies and may result from the disease process necessitating surgical care, from a complication of the surgical procedure and anaesthesia or from comorbidities [8-10]. In LMICs like Nigeria, a three-pronged delay in patients´ care is common namely delay before presentation, delayed referral and in-patient delays from theatre waiting list or operational bottlenecks [9,11-13]. These often-avoidable delays together with limited health facilities and poor infrastructure may contribute significantly to mortality during surgical care in the low HDI nations [8-13].

In summary, among hospital patients, mortality could be due to surgical or co-existing medical condition, unidentified clerical or technical errors, delayed treatment or inadequate health infrastructure [5,8,10]. From the foregoing, the need for periodic audit of pattern of mortality in a specified health system cannot be overlooked bearing in mind the devastating consequences of unregulated, inefficient or totally wrong surgical care to a population served by such health system. As a response to clues drawn from a systematic and critical surgical audit, a unified surgical strategy or modification of an existing management protocol could be adopted or improved to serve the surgical needs of the communities meritoriously.

Nevertheless, there is paucity of data on this subject in our centre. Despite availability of clinical data on mortality pattern in other institutions in Nigeria and Africa, there are no documentation to inform general surgeons working in our region on the current mortality rates, the trend over time and the etiological spectrum of pathologic entities that cause the deaths [5,8,10,12-14]. The aim of this study was to document the incidence and pattern of mortality among general surgery in-patients in our centre. The study also aimed to analyze the impact of some clinical indices on mortality for general surgery patients in our environment.

## Methods

**Design and setting:** this was an observational, retrospective case-control study of the pattern and factors associated with mortality among general surgery admissions managed at Alex Ekwueme Federal University Teaching Hospital, Abakaliki (AEFUTHA) between January 2013 to December 2019. AEFUTHA is a teaching hospital located in Abakaliki Metropolis, Ebonyi State, Southeast geopolitical zone of Nigeria. Data were extracted from the case files of the patients managed under general surgery unit within the study period. We determined the contributions (percentages) of various etiologic diseases to mortality in general surgery unit and analyzed the impact of several demographic parameters on mortality within the study period. We equally assessed the impact of selected clinical and perioperative variables on survivors and non-survivors in both non-traumatic and traumatic causes of generalized peritonitis. A variable follow-up period ranging from one to 16 months was noted in the case files of the cases included in this study.

**Study participants´ characteristics/procedure:** the clinical records from the general surgery theatre operation, ward and accident and emergency registers were retrieved and reviewed. Also included were case files of all the general surgical patients who were admitted into surgical Intensive Care Unit (ICU) and surgical amenity (private) wards. Case files with incomplete data were excluded. Case notes of those who died before detailed clinical assessment were also excluded. Only case files of patients 16 years and above were included. Data extracted included socio-demographic parameters like age, sex, educational status and location of settlement. Clinical diagnoses, mode of admission and type of referral to the hospital were noted and recorded in a proforma. The presence of delay before presentation as well as in-hospital delay before treatment was noted. In the trauma group, the proportions of blunt abdominal trauma (BAT) and penetrating abdominal trauma (PAT) were noted. The effects of clinical and perioperative parameters like ASA scores, status of surgeon and anesthetist, comorbidities, type of pathology, injury mechanism, hourly urine output, volume of suctioned fluid and extra-abdominal injury on mortality were noted and analyzed. The main outcome measure assessed in this study was mortality. All cases of mortalities and their respective clinical diagnoses before death were recorded. In this study, the 'cases' are the mortalities while the 'control' are the survivors. In other to eliminate bias, only in-patient deaths recorded in the case files were considered and no assumptions of deaths of patients who stopped hospital visits were included in the mortality cases. This is because death could result from other causes other than the surgical condition. Considering the retrospective nature of this study and the large fixed available sample of general surgery cases within the study period, consideration of sample size determination was informal. For the quantitative variables, patients with ASA score ≥ III were considered high anaesthetic risk. Also, for the emergency presentation in those with non-traumatic generalized peritonitis, delay 0->24 hours was considered mild, 25-48 hours as moderate and >48 hours as prolonged delay. In the emergency group, hourly urine output after initial resuscitation ≥ 30 ml/hour was considered adequate for renal perfusion while <30 ml/hour was inadequate. Data on age, degree of delay before presentation, ASA score, hourly urine output, type of pathology, preoperative blood transfusion and others were grouped into several ordered categories to aid analysis.

**Reporting:** results were reported according to The Strengthening the Reporting of Observational Studies in Epidemiology (STROBE) guidelines [15,16].

**Data analysis:** data analysis was done with Statistical Package for Social Science (SPSS) version 22.0 (IBM, Chicago, IL, USA, 2015). For the categorical variables, data were summarized in proportions and frequency tables. For continuous variables, we computed the ranges and mean. During analysis, we computed p-values for categorical variables using Chi-square and Fisher´s exact test in accordance with the size of the dataset. Furthermore, we determined the association between some selected clinical variables and mortality using logistic regression analyses. Missing data on hourly urine output, multiple visceral injuries and associated extra-abdominal injuries were addressed using imputed multivariate analyses. Confidence interval was calculated at 95% level and significance at 5% probability level (p<0.05).

**Ethical approval:** the proposal for this study was approved by the Ethics and Research Board of our hospital before commencement of data collection. The approval number is FETHA/AI/AD/05/216.

## Results

**Figure 1:**
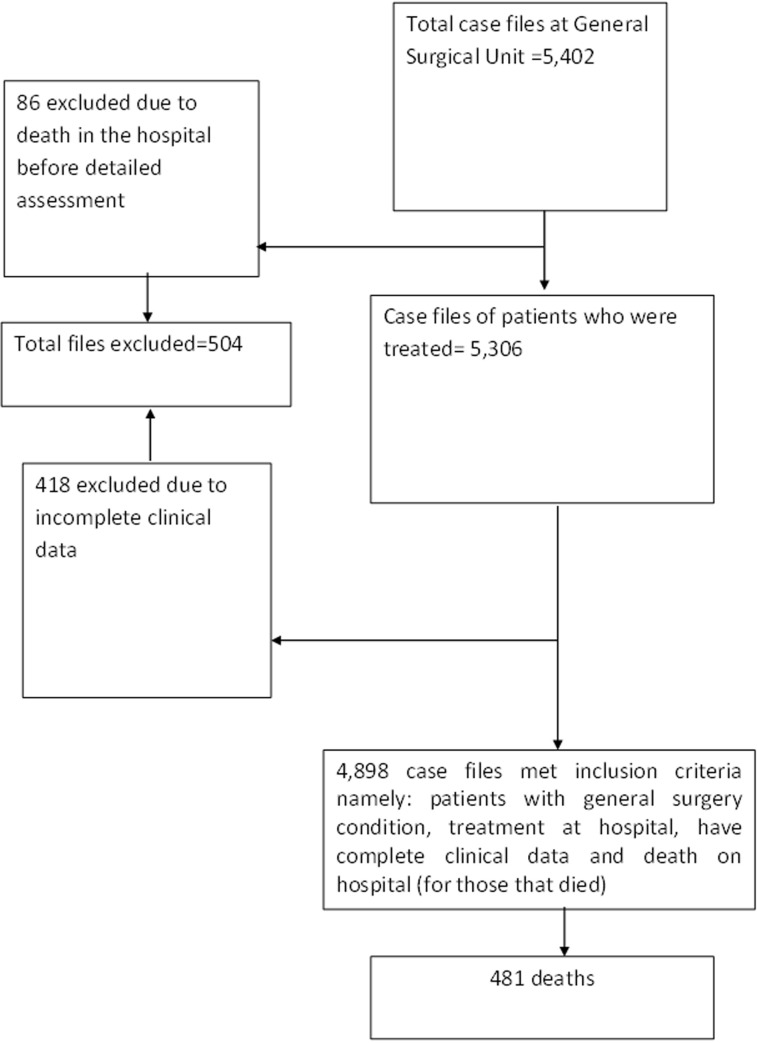
flow diagram of patients’ inclusion and exclusion

**Characteristics of the study participants:** during the period under review, there were 5,402 adult general surgical admissions from the surgical wards, accident and emergency room, amenity wards, theatre and ICU records. However, 504 (9.2%) case files were excluded due to incomplete data or deaths before detailed assessment. The remaining 4,898 cases formed our study population. A flow diagram is shown below (Figure 1). There were 2,988 (61.0%) males and 1,910 (39.0%) females. Overall, their ages ranged from 16-98 years with a mean of 44.73 ± SD 12.38. During the study period, there were 481 deaths giving rise to an overall death per admission crude mortality rate of 9.8%. Of the 481 deaths, 328 (68.2%) were males and 153 (31.8%) were females. Therefore, the overall crude mortality rate for males and females were 11.0% and 8.0% respectively. The male to female ratio for the 481 deaths was 2.1: 1. Though the number of deaths were highest in the 16-45 years age range, mortality rate was highest in the >65 years age group (18.9%). The ages of the dead patients ranged from 18-97 years with a mean of 41.36 ± SD 18.64. More than half (277, 58.0%) of the deaths occurred in patients below 50 years. Majority of deaths were from rural dwellers (314, 65.3%) and those with educational qualification below tertiary level (398,82.7%). Among the 481 deaths, 141 (20.3%), 114 (23.7%) and 96 (20.0%) were farmers, traders and artisans respectively. In addition, 20 (4.2%), 26 (5.4%) and 84 (17.8%) were professionals, students and others respectively.

**Clinical presentation:** majority of the deaths occurred in patients with generalized peritonitis (38.9%), abdominal trauma (16.8%) and gastrointestinal cancers involving the colon, liver, stomach, pancreas, small bowel and biliary tract (10.2%). On the basis of site-specific cancer deaths, breast cancer was the most frequent cause representing 8.3% of all deaths within the study period. The relationship between various aetiologic diagnoses and number of deaths were shown in Table 1. Of the 4,898 case notes evaluated, more than a third, (1,998, 40.8%) presented as emergencies while approximately one-fifth (986, 20.1%) harbored comorbidities ranging from hypertension, diabetes mellitus, benign prostatic hyperplasia, chronic bronchitis, HIV/AIDS, congestive cardiac failure, chronic renal disease and chronic diver disease. The demographic and clinical factors associated with mortality were shown in Table 2. In those managed for non-traumatic generalized peritonitis, delayed presentation beyond 24 hours was present in 1,271 (86.7%) of 1,466 patients that were admitted for generalized peritonitis. Mortality rate was highest in patients with typhoid perforation (26.4%) compared to other causes of generalized peritonitis (OR: 5.33; CI: 2.82-18-32; P=0.001). The impact of other clinical and perioperative indices on mortality is shown in Table 3. In the trauma group, several clinical variables impacted on mortality (Table 4). Overall, over four-fifth (3,998, 81.6%) of the patients engaged in the following deplorable health-seeking behaviors: self-medication, treatment by herbalists, treatment by patent medicine dealers or managed at prayer homes before presenting to the specialist general surgeon.

**Table 1:** common etiological causes of death

Clinical variables	Number of deaths	Percent (%)
Generalized peritonitis	187	38.9
Intestinal obstruction	64	13.3
Abdominal trauma	81	16.8
Breast cancer	40	8.3
Gastrointestinal cancers	49	10.2
Other malignancies	21	4.4
Thyroid diseases	2	0.4
Other benign diseases	37	7.7
Total	481	100.0

**Table 2:** demographic and clinical determinants of mortality rates

Clinical parameter	Number of patients	Number of deaths	Mortality rate (%)	χ2(p-value)	Odd ratio (95% CI of odd ratio)
**Age (years)**					
16-45	2,936	239	8.1	7.2(0.016)	3.38 (1.48-8.24)
46-65	1,307	118	9.0		
>65	655	124	18.9		
**Comorbidities**					
Present	986	122	12.4	5.3*(0.044)	2.94 (1.16-7.33)
Absent	3,912	359	9.2		
**ASA class**					
ASA I-II	1200	29	2.4	12.1(0.002)	13.73 (3.67-21.19)
ASA III	2,404	231	9.6		
ASA IV	1,226	180	14.7		
ASA V	68	41	60.3		
**Mode of admission**					
Emergency	1,998	376	18.8	25.3(0.000)	18.42 (5.66-34.52)
Elective	2,900	105	3.6		
**Status of surgeon**					
Surgeon‡	948	84	8.9	5.8(0.061)	1.86 (0.48-32.61)
Trainee	3,062	337	11.0		
NOM	888	60	6.8		

ASA: American society of anaesthesiologists; NOM: non operative management; CI: confidence interval; surgeon‡: board certified, *Fisher's exact test used

**Table 3:** predictors of mortality for non-traumatic generalized peritonitis

Clinical indices	Number of patients	Number of deaths	Mortality rate (%)	χ2(p-value)	Odd ratio (95% CI of odd ratio)
**Type of pathology**					
Typhoid perf	280	74	26.4	13.7 (0.001)	5.33 (2.82-18.32)
PUD perf	461	48	10.4		
Rup. append	511	40	7.8		
Others	214	25	11.7		
**Delayed presentation (hours)**					
0-24	195	10	5.1	11.3(0.003)	9.73 (1.21-10.26)
25-48	501	49	10.0		
>48	770	128	16.6		
Volume of suctioned fluid (L)					
<4	1,074	129	12.0	*5.8(0.044)	1.37(3.92-12.14)
>4	360	57	15.8		
NOM	32	1	3.1		
**Status of anaesthesiol**					
Specialist	634	76	12.0	3.8(0.071)	2.18(0.41-17.18)
Non-specialist	800	110	13.8		
NOM	32	1	3.1		
Hourly urine output after initial resusc. (ml)					
≥30	1,030	120	11.7	6.3(0.036)	11.35(1.89-22.51)
<30	392	64	16.3		
No record	44	3	6.8		

PUD: peptic ulcer disease; perf: perforation; NOM: non-operative management; C.I: confidence interval; Rup: ruptured; Append: appendix; resusc: resuscitation; *Fisher's exact test used; Anaesthesiol: anaesthesiologist

**Table 4:** predictive indices of mortality in trauma patients

Clinical features	Number of patients	Number of deaths	Mortality rate (%)	χ2 (p-value)	Odd ratio (95% CI of odd ratio)
**Mechanism of injury**					
Blunt trauma	446	56	12.6	4.6(0.064)	4.12(0.28-7.44)
Penetrating	276	25	9.1		
**Multiple visceral injury**					
Yes	173	32	18.5	8.4(0.008)	9.33(8.41-28.22)
No	433	42	9.7		
No record	116	7	6.0		
**Degree of delay before surgery (hours)**					
OM after 48 hrs	504	70	13.9	*18.6(0.001)	1.88(3.13-19.46)
OM within 48hs	110	7	6.4		
NOM	108	4	3.7		
**Associated extra-abdominal injury**					
Yes	84	13	15.5	7.8(0.005)	12.27(4.43-18.14)
No	626	68	10.7	7.8(0.005)	12.27
No record	12	0	0.0		
**Multiple blood transfusion (preoperative)**					
Yes	249	41	16.5	6.4(0.021)	1.18(7.28-34.22)
No	473	40	8.4		

OM: operative management; NOM: non-operative management; C.I: confidence interval; *Fisher's exact test used

**Anaesthetic assessment and surgical treatment:** approximately half (49.1%) of the patients had ASA III score, followed by ASA IV (25.0%). Mortality rate was highest in patients with ASA V (60.3%) as shown in Table 2. Of the entire 4,898 case notes evaluated, 888 (18.1%) were for patients managed conservatively while 81.9% received surgical intervention. Of the 888 cases managed non-operatively, mortality was reported in 60 giving a mortality rate of 6.8% in this group. The rest of the deaths (421, 87.5%) were in those managed operatively, giving a mortality rate of 10.5% for the 4010 patients managed operatively. Majority of the mortalities occurred in cases performed by trainee surgeons (72.9%) compared to cases done by board certified surgeons (27.1%).

**Mortality indices:** generally, age above 45 years, high ASA scores and emergency admission were significantly associated with mortality (Table 2). For patients with non-traumatic generalized peritonitis (Table 3), there was statistically significant difference in mortality rates among patients with typhoid perforation (p=0.001), delayed treatment (p=0.003) and in those with suctioned peritoneal fluid > 4 litres (0.044). In non-traumatic peritonitis, data on hourly urine output were missing in 44 (3.0%) files; three (6.8%) deaths were found in those with missing files (Adjusted Odd Ratio (aOR): 11.39; CI: 1.92-22.60). In the trauma group (Table 4), multiple visceral injuries (p=0.008), delayed operative treatment (p=0.001), associated extra-abdominal injuries (p=0.005) and preoperative multiple transfusion requirements (p=0.021) were significantly associated with mortality. In this group, data on “multiple visceral injuries” were missing in 116 (16.1%) case notes; seven deaths (6.0%) were noted among those with missing data (aOR: 9.46; CI: 8.49-28.32). Similarly, no information on “associated extra-abdominal injury” was found on 12 (1.7%) of 722 patients with abdominal trauma. There was no mortality recorded among the 12 patients (aOR: 12.24; CI: 4.41-18.11).

## Discussion

Majority of deaths were due to generalized peritonitis (38.9%), cancers (22.9%) and abdominal trauma (16.8%). Though ruptured appendix accounted for majority (511, 34.9%) of cases of peritonitis, typhoid perforation was the most lethal with a mortality rate of 26.4% compared to 7.8% for ruptured appendix and 10.4% for peptic ulcer disease (PUD) perforation. In the trauma group, majority were due to BAT (64.5%) with a crude mortality rate of 12.6%; the mortality rate was 9.1% for PAT. Overall, delayed presentation, lower rank of surgeons and anaesthetists, associated extra-abdominal injuries, comorbidities, advanced age >45 years and emergency presentation were associated with increased mortality. Audit of surgical mortality provides an overview of the leading causes of death in patients who require surgical care, thus identifying system or process error and trends in deficiency of care. Existing data showed that surgical mortality audit helps develop strategies to reduce deaths in the surgical unit and provides opportunities to inform, educate, facilitate change and improve quality practice in the surgical arena [17-23]. The quality of care in the surgical unit is an important index of assessing the optimum health care delivery pattern in a health institution [17,20,22-25].

In the current study, mortality pattern showed male preponderance with predominantly rural dwellers that comprised mostly subsistence farmers and traders. These findings are similar to previous results from Nigeria [8,10,23], Ethiopia [12], Tanzania [14], Malawi [26], Ghana [19] and Uganda [13]. This observation of higher mortality tolls on residents of rural settlements have been found to be related to lower access to timely and safe surgery and anesthesia care that has become a recognized barrier to good surgical outcomes in LMICs [2,4,8,11,18,22,27].

In the current discourse, 68.4% of mortality cases from generalized peritonitis presented after 48 hours of onset of symptoms. In Ghana, similar degree of late presentation was reported among 346 general surgical patients [19]. The authors found that 60% of mortality were from late presenters, who were mostly referrals from other hospitals [19]. Published data showed that late presentation of surgical patients is phenomenal in sub-Saharan Africa (SSA) especially in district hospitals due to multi-faceted economic, social and religious factors [2,4,18,21,25,26,28].

Despite the recommendation by the Lancet Commission on Global Surgery and the World Bank that basic emergency and essential general surgical services should be available at all district hospitals, the delivery of essential surgery package at district centres is often a challenge in LMICs especially SSA [2,4,23,27,29]. Generally, patients living in remote rural or semi-urban areas in LMICs encounter difficulties accessing safe surgical and anesthesia care due to poor or deplorable road network, inadequate diagnostic and therapeutic tools, inadequately trained personnel and limited infrastructure in the district health facilities [5,13,23,27-29]. Sub-Saharan Africa bears a disproportionately higher burden of morbidity and mortality from surgical conditions compared to other regions with approximately 35.7-99.4% of the population unable to access surgical care [26].

Unfortunately, these factors in synergy with late presentation contribute to poor patient outcomes even when they are finally managed in tertiary hospitals [26,28-32]. In patients with non-traumatic peritonitis, we found that crude mortality rate was disturbingly high to the tune of 16.6% in patients who presented after 48 hours of symptoms compared to a rate of 5.1% for those admitted within 24 hours of onset of problems. Indeed, the danger lies with the delay and not in the operation [4,9,17,19,26,28,33]. Published data from Nigeria [9,18,24,28], Uganda [13], Cameroon [33], Ghana [19], Malawi [26] and Ethiopia [12] overlapped with the above findings. In SSA, the delay is worrisome and comprised serial delays occasioned by late presentation, prolonged outpatient assessment and investigation time, in-hospital delays before treatment and lastly but the most annoying, in-theatre delays occasioned by incessant electricity outage, unsterilized linens and instruments, limited peri-operative nursing and anesthetic workforce, faulty electrosurgical unit, theatre lights and other operating instruments and lack of essential theatre supplies and anesthetic drugs. Regrettably, the above barriers to prompt surgical services were very prominent in this study and other similar studies in Nigeria [8,10,18,23,24,27,28], Uganda [13], East Africa [5], Kenya [22], Malawi [26], Rwanda [29], Cambodia [30], Ghana [19] and in multinational studies from Africa [21] and LMICs [20].

The high mortality rate of 9.8% observed in this study is worrisome, but highlights the impact of the several barriers to safe and essential surgical and anaesthesia care earlier pointed out in this discussion. Remarkably, more than a third (40.8%) of the general surgical patients presented in emergency, which no doubt contributed to high mortality rate recorded in this study. Available data showed that under emergency set up, pre-operative preparation is suboptimal, patient physiologic reserve is diminished, usage of WHO safe surgery checklist is significantly reduced, diagnostic work up is limited and schedule of the surgery may be at night hours when lower rank of surgeons may be available to perform the operation [6,21,28,31,32].

In rural Ghana, a comparable mortality rate of 11.5% was quoted for patients who underwent emergency exploratory laparotomy [19]. The higher mortality rate reported from Ghana may be explained by the fact that all the cases were managed under emergency set up, which is known to have higher morbidity and mortality compared to cases treated under elective circumstances [19]. Another reason for the higher mortality rate in Ghana may be related to the delayed presentation and treatment as the authors recorded a mortality rate of 13.5% for referred cases that presented late [19]. Also, the authors reported on perioperative mortality rate (POMR) which traditionally incorporates cases with documented higher operative and anaesthetic risks and subsequently higher mortality indices compared to an ‘overall crude mortality rate´ of surgical admissions that was reported in our current study.

Similarly, Ogbuanya and Ugwu working in Southeast Nigeria reported on a large series of 879 general surgery patients who underwent emergency laparotomy at district hospitals and recorded a mortality rate of 10.6% [28]. The higher rates in Ghana [19] and Southeast Nigeria [28] where only emergency cases were analysed suggest increased risk of death under emergency set up. Furthermore, mortality rate in the emergency group in the current review was 18.8% compared to 3.6% in the elective group (OR: 18.42; CI: 5.66-34.52; P=0.000). It has been shown that emergency presentations especially acute abdominal conditions initiate systemic disturbances that often overwhelm the patients´ immune mechanisms which subsequently predispose to dangerous rates of morbidity and mortality [19,28,33,34].

We found that comorbidities were present in about a fifth (20.1%) of all case files reviewed and that the crude mortality rate was 12.4% for those with comorbidities akin to reports from previous studies [8,10,11,19,23,24]. African patients are unique in their health-seeking behaviors with many often not being aware of their comorbid medical conditions when they present in emergency [9,11,18,19,21]. Unfortunately, most derangements arising from cardiovascular, renal, metabolic and endocrine comorbid conditions cannot be optimized in emergency setting before surgical treatment and have led to devastating perioperative outcomes including death on table or in the operating theatre [10,11,18].

In the classic manner, we observed that death from intra-peritoneal sepsis, cancers, abdominal trauma and intestinal obstruction took the highest tolls and these resulted overlapped with observations made in Rwanda, India, Nigeria and Uganda [8,13,17,19,28,29]. The reasons adduced for higher rates of deaths in the laparotomy and oncologic procedures included high septic complications and overall poor prognosis of cancer cases respectively [13,17,19,28,29]. Nevertheless, we observed that early presenters especially younger patients without comorbidities had impressive outcomes, validating the time-honored fact that the danger lies with the delay and not in the surgical operation [4,9,17,19,26,28,33].

We observed a number of statistically significant relations between several demographic, clinical and perioperative factors and mortality index. On the patients´ demographics, age above 45 years was associated with increased death, perhaps due to co-existing medical illnesses, diminished physiologic reserve and higher incidence of cancer and metabolic diseases in this age group. Data culled from India, Nigeria and other parts of Africa support the above observation [8,10,11,13,17,21,24,28]. Moreover, comorbidity, type of pathology and extent of disease process represent important indices of ASA scores which proportionally correlates with mortality rates.

In the group managed for non-traumatic peritonitis, the key determinants of mortality were typhoid perforation as a cause of perforation peritonitis, delayed presentation or treatment, high volume of intra-peritoneal collection and persistently low hourly urine output (Table 3). The high propensity of virulent salmonella organisms to initiate systemic toxicity and generalized multi-organ failure has been described and well documented [28,33,34] and may have contributed to the high mortality associated with typhoid perforation in this study. In immune compromised patients with generalized peritonitis and those with pre-existing renal, cardiac or hepatic derangements, end-organ failure may be accelerated with progressive adverse clinical course [33,34].

In the trauma patients, non-operative management and no requirement for multiple blood transfusion were associated with least mortality rates (Table 4). This observation supports the recent change in surgical principle that favors non-operative management of solid organ injuries as against operative treatment [29,31]. Other main independent predictors of mortality in this group included multiple visceral injuries and associated extra-abdominal injuries which perhaps, added to the volume of blood loss, degree of intra-peritoneal soilage, shock and high degree of metabolic response to trauma. Moreover, evidence from published clinical studies indicates that multiple injuries predispose to immobility with subsequent higher risk of deep vein thrombosis and for operative cases, increased operation time and extent of procedure [31,33,34].

This study presents two main strengths. First, to the best our knowledge, this research work provides a pioneer report on general surgery mortality in Nigeria. Second, this study comprised the largest series of surgical mortality cases in Southeast Nigeria and the foremost scientific documentation on factors that affect mortality rates among surgical patients in southeast geopolitical zone of Nigeria.

**Limitation:** there were a number of limitations in this study. The main limitation being that it is a single centre study and therefore mandates an indigenous multi-centre research work to validate our figures and compare scientifically with data from other nations or regions. In addition, the retrospective nature of this study did not provide ample opportunity for elaborate information especially on outcome measures that contributed to increased mortality. Due to the retrospective nature of the study, planned follow up was lacking and this seriously limited the findings of this study. More so, extrapolation of our findings to other studies on surgical mortality in tertiary hospitals in Nigeria and other African countries should be done with caution as ours presented only mortality audit in general surgery and not mortality for surgical admissions.

## Conclusion

Mortality in the general surgery unit is still significantly high in our environment. This is predicated on several clinical, pathologic and perioperative factors particularly late presentation in patients with generalized peritonitis, abdominal trauma and cancers. The mortality rate was higher in those with generalized peritonitis, cancer and age 45 years and above. Comorbidities, emergency presentation and high ASA scores were significantly associated with increased mortality rates. In the non-traumatic peritonitis cases, the main independent predictors of mortality were typhoid perforation, delayed presentation beyond 24 hrs and persistently low urine output. In those with abdominal trauma, need for operative management, multiple visceral injuries, associated extra-abdominal injuries and multiple blood transfusion requirement represented the main independent predictors of mortality.

**Recommendation:** greater public enlightenment and campaign on subjects like cancer screening, prevention and early treatment, road safety laws and injury prevention are expedient in our environment. The health implications of poor personal hygiene, the elegance of early presentation to hospitals in terms of outcomes and increased awareness of the value of routine medical examinations cannot be overemphasized and should be promoted and raised to public health domain. Improved national health budget, provision of pipe born water, implementation of safe sanitary disposal, wider coverage of National Health Insurance Scheme (NHIS) and construction of good road networks by the government are salutary.

### What is known about this topic

- Late presentation with delayed treatment of surgical patients who often have advanced surgical diseases is phenomenal in Africa and other LMICs;
- Emergency and essential surgical and anesthesia services are grossly inadequate in developing nations especially sub-Saharan Africa due to poverty, low workforce, poor road networks and limited diagnostic and therapeutic facilities;
- Despite being younger with a lower surgical risk profile and undergoing fewer complex surgeries, patients in Africa are twice as likely to die after surgery when compared with outcomes at the global level.

### What this study adds

- The first national research project on the subject “mortality audit in general surgery unit” in Nigeria;
- The largest regional series of surgical mortality in Southeast Nigeria and the first scientific report on surgical mortality in Ebonyi Province of Southeast Nigeria;
- The first regional scholarly report that evaluated the impact of socio-demographic and clinical variables on mortality rates among surgical admissions in Southeast Nigeria.
